# The Benefits of Integrative Medicine for Pain Management in Oncology: A Narrative Review of the Current Evidence

**DOI:** 10.7759/cureus.41203

**Published:** 2023-06-30

**Authors:** Victoria Carvalho, Shahid B Rangrej, Rajni Rathore

**Affiliations:** 1 Medical School, Saint James School of Medicine, Arnos Vale, VCT; 2 Anatomy/Research, Saint James School of Medicine, Arnos Vale, VCT; 3 Pharmacology and Therapeutics, Saint James School of Medicine, Arnos Vale, VCT

**Keywords:** oncologic pain, cancer pain management, chronic pain, pain, integrative medicine

## Abstract

The aim of this evidence-based study is to narrate and evaluate the current evidence on recommendations for practicing physicians and other healthcare providers regarding integrative approaches to managing pain in patients with cancer. This review will assess the guideline recommendations and analyze the role of integrative medicine in addressing cancer pain in patients. The literature search highlights relevant studies that will inform evidence-based recommendations for practicing physicians, highlighting their relevance and weaknesses. Acupuncture, massage, and hypnosis have intermediate-strength evidence quality and are moderately recommended for various types of cancer pain. Most of the evidence points to acupuncture being recommended for aromatase inhibitor-related joint pain, hypnosis for procedural pain, and massage for palliative care pain. Other practices with lower-quality evidence include yoga and guided imagery with progressive muscle relaxation, mostly recommended for general cancer pain or musculoskeletal pain. Additionally, music therapy is recommended for procedural or surgical pain. Low-quality or inconclusive evidence was found for other mind-body interventions or natural products. Similarly, there is insufficient evidence to provide recommendations for pediatric patients. Further research is required to enhance our understanding of the role of integrative medicine interventions in caring for cancer patients.

## Introduction and background

According to the American Cancer Society (ACS), oncologic pain derived from cancer itself can vary according to a series of factors, including the type of cancer, the stage, pain tolerance of the patient, and concomitant conditions [1]. Other factors, including lifestyle choices, such as drinking coffee, have been linked to the suppression of carcinogenesis in breast tissue [2]. A suppression in carcinogenesis may prevent the progression of cancer to a more advanced stage, and a higher stage has been linked to a higher pain prevalence [3]. A 2007 meta-analysis concluded that 64% of the studied cancer patients with advanced/metastatic/terminal cancer reported pain, compared with 53% of patients with all cancer stages [3]. Hence, lifestyle factors might play a role in oncological pain.

In the same meta-analysis [3], the authors investigated the pain prevalence in cancer patients. Researchers found that when comparing gynecological, urogenital, breast, lung/bronchus, and gastrointestinal cancers, all showed a pain prevalence in the 52% and 60% range. Head/neck cancers were found to have the highest pain prevalence of 70%. Therefore, indicating a different pain prevalence in different types of cancers.

Integrative practices, such as acupuncture, massage, and yoga, have demonstrated positive outcomes in the management of chronic pain [4]. Heather Greenlee, the co-chair of the Society for Integrative Oncology (SIO) Clinical Practice Guideline Committee, stated in a press release from the SIO, "Pain is a clinical challenge for many oncology patients and clinicians, and there's a growing body of evidence showing that integrative therapies can be useful in pain management. However, clear clinical guidance regarding the appropriate use of these approaches has been lacking" [5]. In December 2022, a new joint practice guideline from the SIO and the American Society of Clinical Oncology (ASCO) was published [6].

The European Pain Federation (EFIC) defines pain as an unpleasant sensory and emotional experience that may or may not be associated with tissue damage [7]. Chronic pain, which lasts longer than three to six months, is a debilitating condition with significant socioeconomic implications. While pharmacologic approaches have traditionally been used for chronic pain treatment, concerns about opioid prescriptions for chronic non-cancer pain, including issues of tolerance, dependence, and addiction, have prompted increased interest in integrative medicine strategies as alternative pain management options [8].

Integrative medicine is a patient-centered approach that allows individuals to receive a non-traditional course of treatment, facilitating the healing process. This approach combines therapeutic methods from both conventional and alternative medicine, emphasizing the importance of therapeutic relationships. Therapeutic relationships surrounding addressing the needs of the mind, body, and spiritual needs, while still coordinating care with a team of physicians [9]. A good example of the implementation of integrative medicine is the use of acupuncture for chemotherapy-induced peripheral neuropathy [6]. Chemotherapy, in this case, is the conventional medicine approach in conjunction with acupuncture as a form of alternative medicine.

A cross-sectional survey conducted at a tertiary care hospital in India explored physicians' perceptions of complementary and alternative medicine. The findings revealed that 27% of physicians recommended integrative practices for chronic pain patients, with yoga and meditation being the most commonly recommended approaches (23% and 19%, respectively). However, the study also identified significant resistance among physicians toward integrative medicine. Approximately 39% disagreed with using integrative practices even when allopathy was ineffective, and around 60% disagreed with including integrative practices in medical curricula or training doctors on it. Furthermore, 40% of physicians did not recommend complementary and alternative medicine [10].

Integrative medicine takes into account the patient's immediate health and needs, as well as the long-term and intricate interplay between biological, behavioral, psychosocial, and environmental factors [11]. For example, a South Korean case-control study compared the outcomes of patients managed with either Western only or integrative medicine for acute strokes. The study revealed that integrative medicine patients experienced slightly higher costs compared to those who only received Western medicine. However, integrative medicine patients had reduced all-risk mortality rates at three and 12 months after discharge, potentially preventing additional costs from future admissions [12].

Similarly, a retrospective study conducted in Texas showed that patients who received integrative medicine were more likely to experience reduced pain, leading to approximately a 4% decrease in hospital costs [13]. These findings have implications not only for care providers but also for stakeholders, as research suggests that integrative medicine can enhance patient care and outcomes while ultimately reducing hospital costs.

Over 50% of oncology patients experience pain, regardless of cancer stage, and in more advanced cancers, pain prevalence tends to be higher [3]. Combined with the limitation of conventional medicine, which often prescribes opioid analgesics that can cause dependence, or more invasive techniques such as nerve blocks, intrathecal pumps, neuromodulation, and neuroablation, to manage cancer pain [7]. Integrative medicine is a welcomed alternative and when used correctly can result in significant pain reduction and therefore improve the quality of life for cancer patients.

Hence, this review aims to describe the benefits and applications of integrative medicine in the management of oncologic chronic pain. The discussion will focus on integrative medical therapies, such as acupuncture, hypnosis, yoga, and massage therapy, highlighting their potential advantages in the field of oncology.

## Review

Integrative medicine therapies in oncologic pain

Integrative oncology is a patient-centered field that incorporates evidence-based interventions, including mind and body practices, natural products, and lifestyle modifications from various traditions, alongside conventional treatments, to inform cancer care [6].

The ASCO acknowledges that scientific evidence supports the use of therapies such as acupuncture and massage therapy in improving the quality of life for cancer patients and reducing cancer-related pain [14].

This study aims to examine the existing evidence on integrative therapies, including acupuncture, massage, hypnosis, and yoga, in the management of oncologic pain.

Current guidelines

The SIO and the ASCO collaborated to develop a new practice guideline aimed at providing evidence-based recommendations on integrative medicine approaches for managing pain in cancer patients. In total, 227 relevant studies were analyzed to support the development of these guidelines [6]. To develop these practice guidelines, an international multidisciplinary expert panel was assembled, which included a patient representative and a health research methodologist. A systematic review of 1,346 articles from PubMed (1990-2021) and the Cochrane Library (1990-2021) was conducted. The review focused on studies that included adults and pediatric patients experiencing pain during any stage of their cancer care trajectory. It also considered integrative interventions such as acupuncture, acupressure, mind-body therapies, and natural products. The primary outcome of interest in the reviewed articles was pain intensity, reduction, or change in symptoms [6]. After applying the selection criteria, 227 articles formed the basis for the guideline recommendations. However, the panel acknowledged that many primary studies included in the review had limitations and flaws in their study design. Therefore, they relied on systematic reviews to identify relevant primary studies. Limitations in the primary studies included incomparable control arms, lack of blinding, small sample sizes, high attrition rates, and limited statistical power. These limitations decreased confidence in the findings of some interventions. Among the integrative medicine strategies, acupuncture, hypnosis, and massage therapy had the highest quality of supporting evidence for managing cancer pain, as indicated by the number of supporting studies [6]. Tables 1, 2 provide a summary of the recommendations and the number of articles that informed these recommendations for the mentioned integrative medicine methods [6].

**Table 1:** Society for Integrative Oncology–American Society of Clinical Oncology guideline recommendations for adult oncology patients with intermediate quality of evidence and moderate strength of recommendation.

Integrative therapy	Recommendation	Number of studies that informed recommendations
Acupuncture	Aromatase inhibitor-related joint pain	9
	General cancer pain or musculoskeletal pain	9
Hypnosis	Procedural or surgical pain	10
Massage	Non-specified massage for palliative care pain	10
	Reflexology and acupressure for general cancer pain or musculoskeletal pain	7

**Table 2:** Society for Integrative Oncology–American Society of Clinical Oncology guideline recommendations for adult oncology patients with low quality of evidence and weak strength of recommendation.

Integrative therapy	Recommendation	Number of studies that informed recommendations
Acupuncture	Chemotherapy-induced peripheral neuropathy	10
	Procedural pain	9
Yoga	General cancer pain or musculoskeletal pain	4
	Aromatase inhibitor-related joint pain	1
Massage	Non-specified massage for general cancer pain or musculoskeletal pain	4
	Reflexology or acupressure for chemotherapy-induced peripheral neuropathy	2
	Acupressure for procedural pain	3
Guided imagery with progressive muscle relaxation	General cancer pain or musculoskeletal pain	4
Music therapy	Procedural or surgical pain	3

According to the joint panel, the benefits of the 13 recommendations outweigh the risks for patients. However, certain practices such as mind-body interventions, natural products (including omega-3 fatty acids), music therapy, and topical pure emu oil did not meet the criteria for inclusion in the guideline. Additionally, there was insufficient evidence to make recommendations for pain management in pediatric cancer patients.

The guideline also acknowledges that disparities exist among patient groups, which can result in limited access to medical care. Factors such as race and ethnicity, age, socioeconomic status, sexual orientation and gender identity, geographic location, immigrant status, and insurance access can impact the extent to which patients can receive integrative medicine. It is important for researchers to continue working on these interventions in patient care to address and overcome these structural barriers.

Acupuncture

Acupuncture is a technique from traditional Chinese medicine that involves inserting thin steel needles at specific points on the body known as acupoints [15]. It has been found to be beneficial in treating conditions such as overactive bladder, psoriasis, and physical and emotional pain. Acupuncture works by stimulating blood vessels, mast cells, and nerve fibers in the acupoints, leading to the release of corticosteroids and endogenous opiates, such as dynorphin, endorphin, and encephalin, which contribute to its pain-relieving properties [16]. Compared to other pain management methods like pharmaceuticals, acupuncture is considered to have rare and infrequent serious side effects [17].

According to the ASCO, acupuncture has been shown to relieve pain and reduce other cancer-related symptoms such as chemotherapy-induced nausea, vomiting, hot flashes, and dry mouth. However, more research is needed to establish a relationship between acupuncture and other symptoms like anxiety, loss of appetite, and fatigue [14]. In a meta-analysis that evaluated the impact of different traditional Chinese medicine techniques on symptom management and quality of life in cancer patients, acupuncture was found to have a varying degree of effectiveness in alleviating adverse symptoms such as pain, fatigue, sleep disturbance, and gastrointestinal discomfort. The effects of acupuncture were observed to range from small to large improvements in these symptoms [18].

In a new joint practice guideline from SIO and ASCO, four recommendations (1.1, 1.3, 1.8, and 1.11) are provided for the use of acupuncture in managing cancer pain [6].

Aromatase inhibitors are frequently used as adjunctive hormone treatment for breast cancer patients, and have arthralgia as a major side effect [19]. Recommendation 1.1 states that acupuncture should be offered to breast cancer patients experiencing aromatase inhibitor-related joint pain. The guideline is informed by a significant study involving 260 breast cancer patients who were randomized to receive true acupuncture, sham acupuncture, or no acupuncture. The true acupuncture group reported a greater reduction in pain intensity compared to the sham acupuncture group [20].

Recommendation 1.3 suggests the use of acupuncture for general cancer pain or musculoskeletal pain. This recommendation is supported by a study involving 360 patients who were assigned to receive electroacupuncture, auricular acupuncture, or usual care. Electroacupuncture was found to significantly reduce pain intensity compared to auricular acupuncture [21].

Two other recommendations (1.8 and 1.11) with low evidence quality and weak recommendation strength are made for acupuncture. Recommendation 1.8 states that acupuncture can be used for chemotherapy-induced peripheral neuropathy based on a study involving 75 patients, where true acupuncture demonstrated a significant reduction in neuropathic pain [22]. Recommendation 1.11 suggests that acupuncture or acupressure can be used to alleviate pain associated with cancer surgery or other cancer-related procedures. However, more research is needed in this area due to the small sample sizes and the overall low quality of evidence [6]. Despite the small sample size, a 30-patient study selected participants that had undergone a mastectomy and concluded that acupuncture when delivered after the procedure can significantly reduce pain, nausea, and anxiety when compared to usual care [23].

Regarding acupressure, there is some controversy regarding its classification. While some experts consider it a type of acupuncture, others consider it a type of massage. Acupressure involves applying pressure with hands to specific acupoints without the use of needles [24]. For the purpose of this review, it will be considered a type of massage.

Massage

Massage therapy is a tissue manipulation technique where muscles and other soft tissues are moved, rubbed, and pressed. According to the ASCO, this type of therapy shows short-term benefits for chronic pain relief. ASCO further adds that other benefits of massage include decreasing tension and stress levels, easing anxiety and depression, and helping with sleep problems and fatigue [14]. Massage is seen as a non-invasive, cost-effective method that can be a great tool for the pain management of oncology patients [25].

The findings of a meta-analysis involving 559 participants from 12 different studies indicate that massage therapy offers significant relief for cancer pain compared to no massage treatment or conventional care. This therapy has demonstrated effectiveness, particularly in addressing postoperative pain. Among the different types of massage studied, foot reflexology was found to be more effective than body or aroma massage [26].

In the guideline published in the Journal of Clinical Oncology, massage therapy is the most recommended type of therapy, with five out of 13 recommendations specifically mentioning massage. These recommendations include a moderate strength of recommendation for the use of reflexology, acupressure, or general massage for general cancer pain or musculoskeletal pain, as well as for patients experiencing pain during palliative or hospice care. Weak recommendations are made for reflexology and acupressure in patients with chemotherapy-induced peripheral neuropathy, as well as acupressure for palliative care [6].

Recommendation 1.13 of the guidelines suggests the use of classic massage as a therapeutic option for patients who are experiencing pain during palliative and hospice care. The quality of evidence supporting this recommendation is considered intermediate, based on 10 studies that include seven randomized controlled trials and three systematic reviews. Both types of studies, i.e., randomized controlled trials and systematic reviews, consistently conclude that massage is an effective therapy for pain management in cancer patients receiving palliative treatment [6].

Typically, randomized controlled trials in this area involve a range of participants, from 21 to 86 individuals. However, it is worth noting one significant study that included 380 patients with various types of advanced cancers who were experiencing moderate-to-severe pain. In this particular study, massage was shown to have immediate beneficial effects on pain and mood among patients with advanced cancer. However, no long-term reduction in pain was observed [27].

Another recommendation, i.e., recommendation 1.5, pertains to a classic massage for cancer patients experiencing chronic pain following breast cancer treatment. Despite the low quality of evidence, this recommendation is classified as moderate. It is based on a total of four studies, including two reviews and two clinical trials [6].

A meta-analysis examining the effects of manual therapy on chronic musculoskeletal pain in the upper limbs and thorax of female breast cancer patients included five randomized controlled trials. The researchers found that manual therapy reduced the intensity of chronic musculoskeletal pain, although no significant difference was observed in quality of life compared to control groups. The study concluded that manual therapy is considered effective for treating chronic musculoskeletal pain in the upper limbs and thorax of female breast cancer survivors [28].

In contrast, a comprehensive review that assessed the effects of massage with or without aromatherapy on pain and other cancer-associated symptoms examined a total of 19 studies (21 reports) with very low-quality evidence, involving a combined total of 1274 participants. When it comes to pain, the quality of evidence comparing the massage group to the no-massage group was deemed very low. This was primarily due to the small size of most studies and their poor reporting, which resulted in an unclear or high risk of bias. Notably, one study even found that short-term pain was greater in the massage group compared to the no-massage group. The review highlighted the insufficient clinical evidence available on the effectiveness of massage for symptom relief in individuals with cancer, emphasizing that most studies were too small to provide reliable conclusions [29].

Despite the conflicting reviews mentioned, a randomized clinical trial involving 230 patients contributes additional evidence supporting the effectiveness of massage for cancer patients. This particular trial employed a randomized, prospective, two-period, crossover intervention design to investigate the effects of therapeutic massage and healing touch in comparison to presence alone or standard care. The results demonstrated that pain ratings were lower after therapeutic massage and healing touch, and the group receiving therapeutic massage also required less usage of nonsteroidal anti-inflammatory drugs. Based on these findings, the study concluded that therapeutic massage and healing touch are more effective than standard care in reducing pain among cancer patients undergoing chemotherapy [30].

The meta-analysis conducted specifically on musculoskeletal pain in breast cancer patients represents the only study focusing on this aspect [28]. This meta-analysis predominantly supports the moderate recommendation 1.5. However, a contradictory review, which included a greater number of low-quality studies, concluded that there is a lack of clinical evidence regarding the effectiveness of massage for symptom relief in individuals with cancer [29]. Nonetheless, the substantial 230-patient randomized clinical trial further underscores the potential benefits of therapeutic massage and healing touch over standard care in reducing pain among cancer patients [30]. It should be noted that this clinical trial does not specifically address patients experiencing chronic pain following breast cancer treatment, as indicated by recommendation 1.5.

Recommendation 1.11 proposes the utilization of acupuncture or acupressure for patients undergoing cancer surgery or other cancer-related procedures. This recommendation is supported by a total of 12 studies, with seven focusing on acupuncture alone and three investigating the effects of acupressure. The overall quality of evidence was categorized as low, primarily due to the limited sample sizes, leading to a weak recommendation [6]. Among the three studies specifically examining acupressure, two of them were randomized clinical trials specifically addressing bone marrow aspiration and biopsy pain. The first trial, which involved a small sample size of 37, demonstrated that acupressure significantly reduced the proportion of patients experiencing severe pain compared to sham acupressure [31]. In the second trial, involving 90 patients, acupressure was found to result in the lowest procedural pain score when compared to sham acupressure or no treatment [32]. The third trial encompassed 60 patients who underwent surgical procedures for gastric cancer, and it revealed significant differences in postoperative pain, with the group receiving postoperative acupressure experiencing less pain in comparison to the control group [33].

Reflexology is a massage technique where pressure is applied to specific reflex points on the hands and feet, corresponding to specific areas of the body. It has been reported that massaging these points can enhance blood supply to related organs and generally reduce anxiety, pain, and fatigue [34]. The guideline recommends reflexology for oncology pain in two instances: recommendation 1.4 for patients experiencing pain during systemic therapy for cancer treatment and recommendation 1.9 for patients with chemotherapy-induced peripheral neuropathy. Although both recommendations also mention acupressure, none of the combined nine supporting studies include acupressure [6]. It is important to distinguish between reflexology and acupressure. As mentioned previously, acupressure uses acupoints that are the same as those used in acupuncture. On the other hand, reflexology uses reflexes that are not related to acupoints, although there might be some overlap between them [34].

Recommendation 1.4 is classified as a moderate recommendation with intermediate-quality strength. It is supported by a total of seven randomized trials involving a combined sample of 1096 patients with diverse types of cancer. With the exception of one trial, all of these studies reached the conclusion that patients who underwent reflexology treatment exhibited significantly reduced pain compared to the control group [6].

The trial that did not show significant pain reduction in patients who received reflexology focused on patients with advanced-stage breast cancer undergoing chemotherapy and/or hormonal therapy. A total of 286 women were randomized into three groups: reflexology (n = 95), lay foot manipulation (n = 95), or conventional care (n = 96). Reflexology was performed by certified reflexologists trained in the Ingham method of reflexology, either at home or in clinical settings. Although no adverse effects were reported, the only benefit experienced by the reflexology group was a statistically significant improvement in physical functioning compared to the control group. Another finding was a reduction in the severity of dyspnea in the reflexology group compared to both the control group and the lay foot manipulation group. However, no significant differences were found between the reflexology group and the other groups regarding breast cancer-specific health-related quality of life, depressive symptomatology, state anxiety, pain, or nausea [35].

This study contradicts the findings of another large-scale trial that also investigated the effects of reflexology in breast cancer patients [36]. The second study involved 256 women with advanced breast cancer undergoing chemotherapy, targeted therapy, and/or hormonal therapy, who were randomly assigned to either a reflexology group or a control group. In this study, reflexology was administered through a home-based intervention delivered by a friend or family caregiver. This intervention successfully reduced patient-reported pain [36]. The disparity between the two studies may be attributed to the level of experience of the reflexologists or caregivers involved, as less experienced reflexologists may yield outcomes similar to lay foot manipulation, as observed in the previous study [35]. Therefore, conducting additional studies involving experienced reflexologists and comparing the effectiveness of reflexology between reflexologists and trained caregivers could provide further clarity on its efficacy for patients with advanced breast cancer.

Another recommendation in the new cancer pain guideline, recommendation 1.9, mentions reflexology or acupressure, despite the lack of supporting studies specifically for acupressure. The only area that has sufficient evidence to support the recommendation is acupressure for procedural pain, as previously discussed in recommendation 1.11. Recommendation 1.9 is considered to have low-quality evidence, with only two supporting studies, and is classified as a weak recommendation for patients experiencing chemotherapy-induced peripheral neuropathy. Both studies randomized approximately 60 patients into two groups: a control group and a reflexology group. No adverse effects were reported in either study [6]. In the first study, no significant differences were found between the two groups regarding peripheral neuropathy-related pain severity and incidence. However, the reflexology group showed improvements in sensory functions compared to the control group [37]. The second study focused on patients with gynecologic cancers who performed self-foot reflexology. In contrast to the previous study, this study found that self-foot reflexology significantly reduced symptoms of peripheral neuropathy, decreased interference with daily activities, increased peripheral skin temperature levels, and decreased anxiety and depression compared to the control group [38]. The conflicting results highlight the necessity for further research in this area, particularly with larger sample sizes.

Hypnosis

Hypnosis is a technique in which a professional suggests that a person undergoes changes in sensations, perceptions, thoughts, or behavior. It has been recognized as a valuable medical tool, with studies suggesting its benefits for pain management and the treatment of phobias, depression, dissociative disorders, and psychotic disorders, among others. A review conducted in 2014 found that hypnotic processes can modify both internal (self-awareness) and external (environmental awareness) brain networks. Specifically related to pain, the review indicated that hypnosis can modulate the interconnected network of cortical and subcortical regions involved in processing noxious stimuli, leading to a significant decrease in pain sensation [39].

According to recommendation 1.10, hypnosis can be considered as an option for patients who experience pain during surgical or procedural procedures. This recommendation is supported by 10 studies and is classified as having intermediate-quality evidence, resulting in a moderate recommendation. Among the 10 studies, six specifically focused on cancer patients undergoing breast biopsies, while the remaining studies examined procedures such as transrectal ultrasound-guided prostate procedures, percutaneous tumor treatments, and bone marrow aspirates and biopsies. Out of the 10 studies, nine concentrated on adult patients, with only one study specifically investigating pediatric cancer patients [6]. The pediatric review encompassed seven randomized controlled clinical trials and one controlled clinical trial. Despite certain methodological limitations, the review concluded that hypnosis has the potential to be a clinically valuable intervention for managing procedure-related pain and distress in pediatric cancer patients. All of the reviewed studies consistently reported statistically significant reductions in pain and anxiety/distress [40]. Additionally, a recent umbrella review of meta-analyses, which examined 34 publications, also concluded that hypnosis significantly reduces procedure-related pain and distress. However, it pointed out the small sample sizes and moderate quality of the majority of the studies, underscoring the need for further research in pain management, particularly in pediatric patients, where hypnosis shows promising pain reduction properties [41].

The guideline highlights two well-designed studies that included an attention control group as well as a standard-of-care group. In both of these studies, hypnosis was provided throughout the entire procedure, rather than just shortly before it. These two trials serve as the basis for the moderate recommendation regarding the use of hypnosis in managing pain during procedures [6].

The first study [42] involved 236 women who needed to undergo a large core needle breast biopsy. They were randomly assigned to one of three groups: standard care (76 participants), structured empathic attention (82 participants), or self-hypnotic relaxation (78 participants) during the procedure. The self-hypnotic relaxation group experienced a decrease in anxiety, while the empathic attention group showed no statistically significant change in anxiety compared to standard care. In terms of pain, all three groups experienced a significant increase, but the increase was less pronounced in the hypnosis and empathic attention groups compared to standard care. The researchers concluded that both structured empathic attention and hypnosis can reduce procedural pain and anxiety, with hypnosis providing more powerful anxiety relief [42].

The second study [43] involved 201 patients undergoing percutaneous tumor embolization or radiofrequency ablation. They were randomized to receive standard care (control group), empathic attention, or self-hypnotic relaxation with defined empathic attention behaviors. The hypnosis group experienced significantly less pain and anxiety than the standard care and empathy groups, and they also required significantly fewer pain medication units than patients in the other two groups. The researchers concluded that procedural hypnosis, including empathic attention, can reduce pain, anxiety, and medication use [43].

Yoga

Yoga, an ancient practice, offers numerous mental and physical benefits. There are various types of yoga, with hatha yoga being the most popular. Hatha yoga combines different styles and emphasizes physical movements rather than stillness and meditation. It focuses on breath-controlled exercises called pranayamas, followed by a series of yoga postures known as asanas, and concludes with a resting period called savasana [44].

Yoga incorporates breathing exercises, meditation, relaxation techniques, and poses that stretch and flex different muscle groups. It has been scientifically proven to reduce stress, promote mental calmness, and foster a connection between the mind and body. Generally, yoga improves overall quality of life, mood, and physical well-being. It also enhances physical functioning, the ability to perform daily activities, and the capacity to find meaning in the experience of illness. Additional benefits of yoga include reduced sleep problems, pain, fatigue, inflammation, and nausea. Moreover, it helps boost immune function and regulate stress hormones [14].

The first weak recommendation with low-quality evidence is recommendation 1.2, which suggests offering yoga to breast cancer patients experiencing joint pain related to aromatase inhibitors. This recommendation is supported by only one randomized clinical trial [6]. In this study, 167 breast cancer patients undergoing hormone therapy, including aromatase inhibitors or tamoxifen, were divided into two groups. The control group received standard care, while the yoga group received standard care along with a four-week yoga intervention, consisting of twice-weekly sessions lasting 75 minutes each. Comparatively, the yoga group demonstrated greater reductions in musculoskeletal symptoms, including general pain, muscle aches, and overall physical discomfort, in comparison to the control group [45].

Another weak recommendation for using yoga in pain management among cancer patients is recommendation 1.6, which is supported by four randomized controlled trials with low-quality evidence. This recommendation suggests that hatha yoga may be beneficial for patients experiencing pain after undergoing treatment for breast or head and neck cancer. Two of the clinical trials specifically focused on breast cancer patients, while one involved myeloproliferative neoplasm patients, and the other included head and neck cancer patients.

A study examining yoga for pain management in breast cancer patients involved 42 participants who were randomly assigned to two groups. Group 1 participated in a 10-week hatha yoga exercise program, while Group 2 engaged in a 10-week follow-up program that assessed the intensity of arm and shoulder pain. The yoga group exhibited a significant improvement in pain severity, and this improvement persisted even 2.5 months after the treatment, whereas the control group did not experience any notable difference in pain levels before and after the treatment [46].

In another study focused on the feasibility of yoga for pain management in oncology patients, specifically 63 women with metastatic breast cancer, participants were randomized into either a yoga group or a support group. However, not all participants completed the trial, and post-intervention surveys were received from 87% of the randomized participants. Among the completed surveys, 80% of participants in the yoga group and 65% in the support group reported high satisfaction with the intervention [47].

The sole study related to head and neck cancer was a small clinical trial with two groups, consisting of 40 patients who were randomized into an eight-week hatha yoga intervention group and a wait-list control group. In terms of feasibility, five patients from the yoga group discontinued early, while none did so in the wait-list control group. Efficacy measures indicated potential statistically significant benefits in terms of shoulder range of motion, pain, and anxiety [48].

Overall, the studies that analyzed yoga as an oncology pain management strategy are very limited and primarily focus on feasibility. This is important because yoga involves more active and strenuous participation compared to practices like massage and hypnosis. However, larger studies are still needed to gain a better understanding of the benefits of yoga for cancer patients.

Guided imagery with progressive muscle relaxation

Guided imagery is the practice of using mental visualization to enhance mood and physical well-being. It involves creating mental images that engage the senses, such as sight, hearing, taste, smell, touch, or feeling. Positive guided imagery has been widely employed in cancer care and has shown effectiveness in promoting relaxation, reducing stress, improving mood, boosting the immune system, alleviating pain, and minimizing nausea and vomiting [49].

On the other hand, progressive muscle relaxation (PMR) is a nursing intervention that involves sequentially tensing and releasing different muscle groups while attending to the resulting sensations. This technique can help release muscle tension associated with anxiety and promote mental calmness. PMR has also been utilized in cancer care and has demonstrated effectiveness in addressing fatigue, pain, nausea and vomiting, and anxiety, among other symptoms [50].

Recommendation 1.7, which has weak evidence quality, suggests that guided imagery with PMR may be offered to patients experiencing general pain resulting from cancer treatment. This recommendation is supported by four key studies [6]. The first study was a randomized controlled trial involving 208 patients. Half of the patients received four weekly supervised sessions and daily unsupervised sessions of guided imagery and PMR, while the other half received standard care. The intervention group experienced significantly lower levels of pain and fatigue, as well as a reduced frequency of nausea, vomiting, and retching episodes. The researchers concluded that the combined use of guided imagery and PMR can effectively manage certain symptoms in cancer patients undergoing chemotherapy [49]. Importantly, this combined intervention was found to be effective in reducing oncological pain.

Another larger clinical trial, involving 104 patients, examined the combined effect of guided imagery and PMR. The patients were randomly assigned to two groups: Group A received guided imagery and PMR, while Group B received usual care. The study found that guided imagery and PMR were effective in alleviating pain-related distress in terminally ill cancer patients [51].

A supporting study that validates recommendation 1.7 involved 67 patients in a randomized clinical trial. The patients were assigned to three groups: one group listened to relaxation training via audio tapes, another group received live relaxation training from nurses, and the third group received no relaxation training. The patients who received relaxation training showed significant reductions in subjective pain ratings [52].

In contrast, another small clinical trial did not yield similar favorable results. In this study, 60 patients were randomized into three groups: guided imagery group, relaxation group, and control group. Despite the positive reception from patients, with 90% of them recommending the brief psychological intervention to others, the guided imagery and relaxation groups did not show clinically relevant influences on the postoperative physiological course of elderly patients undergoing conventional resections of colorectal cancer [53].

Overall, the studies supporting the use of guided imagery with PMR as an oncology pain management strategy have limitations, including inconsistencies in the blinding of participants, health professionals, data collectors, and data analysts. Additionally, the limited number of favorable findings contributed to the weak strength of the recommendation [6].

Music therapy

Music therapy encompasses various experiences such as listening to music, singing, playing instruments, or composing music. It is a clinical approach that utilizes music to achieve individualized goals, such as reducing stress, improving mood, and enhancing self-expression [54]. Music therapy has been employed in various healthcare settings to alleviate patient pain, anxiety, and stress [55].

Recommendation 1.12, which has weak evidence quality, suggests that music therapy may be offered to patients experiencing surgical pain from cancer surgery. This recommendation is supported by three studies, although the quality of evidence is considered low [6]. All three studies [55-57] demonstrated a significant effect of music therapy in improving surgical pain scores compared to usual care.

In the smallest study, 30 women undergoing mastectomy for breast cancer were randomly assigned to either the music intervention group or the control group. The study found that patients in the music intervention group experienced a greater decrease in mean arterial pressure, anxiety, and pain from the preoperative period until discharge from the recovery room, compared to women in the control group [56].

Another randomized controlled trial examined the effects of music therapy on pain reduction in patients with breast cancer after radical mastectomy. A total of 120 breast cancer patients were randomly and equally divided into a music intervention group and a control group. Music therapy was found to significantly reduce the total pain rating index score in the music intervention group compared to the control group. The researchers concluded that music therapy has both short-term and long-term positive effects in alleviating pain among breast cancer patients following radical mastectomy [55].

A randomized parallel trial investigated the impact of postoperative intravenous sufentanil patient-controlled analgesia combined with music therapy versus sufentanil alone on hemodynamics and analgesia in patients with lung cancer. Sixty patients were randomized into a music therapy group (receiving sufentanil) and a control group (receiving only sufentanil). Both groups received intravenous patient-controlled sufentanil analgesia. The results showed that the music therapy group had significantly lower systolic and diastolic blood pressure and heart rate scores within 24 hours after surgery compared to the control group. Additionally, the music therapy group required a lower frequency of postoperative analgesia and a lower sufentanil dose compared to the control group. The study concluded that the combined effect of music therapy and sufentanil improves the effects of intravenous patient-controlled analgesia compared to sufentanil alone after lung cancer surgery [57].

Overall, despite the limited number of studies, it can be inferred that music therapy can be an effective tool in pain management for oncology patients. Further research in this area would strengthen these claims and potentially expand on the positive results.

Discussion

None of the evidence presented in the guideline [6] reached a classification higher than intermediate evidence. According to their classification, further research is unlikely to change the direction of the net effect, but it may have an impact on the magnitude of the net effect. High-quality evidence suggests that additional research is highly unlikely to alter either the magnitude or direction of this net effect [6]. The absence of recommendations supported by high-quality evidence highlights the insufficient number of quality studies in the field of integrative medicine for managing oncology-related pain.

Among the 13 recommendations, seven were supported by low-quality evidence, indicating a low level of confidence in the available evidence accurately representing the true magnitude and direction of the net effect. Further research has the potential to modify the magnitude and/or direction of this net effect. All recommendations based on low-quality evidence were classified as weak recommendations, except for recommendation 1.5 [6]. Despite the limited quality of evidence, these recommendations were made because no harmful effects were observed in any of the integrative medicine practices. All recommendations clearly stated that the benefits outweighed the risks.

A weak recommendation is established when there is limited evidence regarding the true net effect, with some consistent results but important exceptions. Concerns may arise regarding the quality of the studies and potential disagreements among panelists. Individual considerations and other factors are taken into account, providing some level of confidence that the recommendation offers the best current guidance for practice [6].

A strong recommendation, which was not included in the guideline, would require a high level of confidence that the recommendation reflects the best practice [6]. Given the scarcity of available studies in this area, achieving such confidence is challenging.

Acupuncture had the highest number of overall studies, with a total of 36 supporting studies. However, massage therapy had the most recommendations, with five out of 13. An interesting observation is the discrepancy between recommendations 1.4 and 1.9 [6]. These recommendations suggest that reflexology or acupressure can be used, but none of the supporting studies mention acupressure.

Recommendation 1.9 is controversial, as acupressure is not mentioned and it only has two very small randomized clinical trials as supporting evidence. This raises questions about the effectiveness of reflexology for patients experiencing chemotherapy-induced peripheral neuropathy from cancer treatment. Therefore, more studies are needed to determine if this recommendation is justified [6].

As demonstrated in a study conducted in a tertiary hospital in India, there is still significant resistance among physicians to recommend integrative medical practices to their patients [10]. Conducting higher-quality studies and finding better ways to inform physicians about the benefits of integrative practices, especially in the pain management of oncology patients, could greatly benefit patients.

## Conclusions

The current guidelines on integrative medicine therapies for managing cancer pain provide recommendations based on available evidence. Acupuncture is moderately recommended for aromatase inhibitor-related joint pain in breast cancer patients, while yoga has a weak recommendation supported by only one study. Acupuncture and reflexology have reasonable evidence for alleviating general cancer pain or musculoskeletal pain, but acupressure lacks supporting studies. Limited evidence supports the use of general massage, hatha yoga, and guided imagery with progressive muscle relaxation for general cancer pain or musculoskeletal pain. Acupuncture and reflexology are options for chemotherapy-induced peripheral neuropathy, with stronger evidence supporting acupuncture. Procedural hypnosis is the strongest recommendation for procedural or surgical pain, and acupuncture, acupressure, and music therapy are also effective strategies. Classic massage is recommended for palliative and hospice care pain. Patients experiencing chemotherapy-induced peripheral neuropathy can consider acupuncture and reflexology as options, with stronger evidence supporting the benefits of acupuncture. No evidence was provided to recommend acupressure for chemotherapy-induced peripheral neuropathy. Mind-body interventions, natural products, music therapy, and topical pure emu oil did not meet the inclusion criteria. Further research is needed to better understand the role of integrative medicine in cancer care, and physician education on its benefits is crucial. In conclusion, further research is necessary to gain a better understanding of the role of integrative medicine interventions in the care of patients with cancer. Additionally, greater efforts should be made to educate physicians about the benefits of integrative medicine for cancer patients.
